# T148. THALAMIC-CORTICAL CONNECTIVITY IN PATIENTS WITH AUDITORY HALLUCINATIONS IN SCHIZOPHRENIA

**DOI:** 10.1093/schbul/sby016.424

**Published:** 2018-04-01

**Authors:** Yulia Zaytseva, Eva Kozakova, Eduard Bakstein, Jaroslav Hlinka, Maya Schutte, Iris Sommer, Jiri Horacek, Filip Spaniel

**Affiliations:** 1 National Institute of Mental Health; 2 Utrecht Medical Centre

## Abstract

**Background:**

The auditory verbal hallucinations (AVH) prevalence among patients diagnosed with schizophrenia is more than 70% (Hugdahl et al, 2008). The brain mechanisms of AVH are not yet clarified. In the currect study, we explore the specific brain connections that are usually associated with perception and reception of the speech, being present in patients with AVHs.

**Methods:**

In total 70 patients with first psychotic episode, 41 with auditory hallucinations and 29 patients who had never experienced hallucinations were included into the study based on the following criteria: non-hallucinated patients were defined using PANSS scores (Hallucinations P3 scored as 1)/hallucinated patients (PANSS P3 score 3). All patients underwent resting MRI scans. We analysed the resting state functional connectivity between temporal cortices including TPJ structures (supramarginal gyrus and angular gyrus, parahypocampal gyrus) and predefined thalamic nuclei that are parts of the auditory pathways, specifically: the medial geniculate nuclei(MGB), the mediodorsal nuclei (MDN), having a big spectrum of connections with the limbic system and the reticular thalamic nuclei (RTN) which is a main source of inhibitory signals (using detailed atlas of thalamus, Morel, 2003).

**Results:**

The analysis revealed the increased connectivity between mediodorsal nucleus L and Hesch gyrus (L, R), pahahippocampal gyrus (L,R) as well as between mediodorsal nucleus (R) and parahipocampal gyrus (L). The decreased connectivity was detected between medial geniculate body (L), Medial geniculate body (R) and inferior parietal lobule (L) as well as between medial geniculate body (L) x parahyppocampal gyrus (L) (FDR corr p=0.05).

**Discussion:**

We confirmed our hypothesis on the altered connectivity between thalamic nuclei and auditory cortex (Heshl gyrus, parahippocampal gyrus) and inferior parietal cortex in patients with AVHs. The findings go in line with other studies on functional dysconnectivity with right hippocampal formation and mediodorsal thalamus compared to patients without lifetime AHs (Diederen et al, 2010; Shinn et al, 2013). The patterns of decreased connections presumably indicate a sensory defect: decreased connectivity between medial geniculate nuclei (main auditory pathway) and parahippocampal gyrus (memory retrieval) and inferior parietal cortex (interpretation of sensory information, Radua et al, 2010). The patterns of increased connections presumably imply alterations of emotional processing (projections from mediodorsal nuclei to auditory cortex) and deserve further investigation.

